# Wax‐Transferred Hydrophobic CVD Graphene Enables Water‐Resistant and Dendrite‐Free Lithium Anode toward Long Cycle Li–Air Battery

**DOI:** 10.1002/advs.202100488

**Published:** 2021-06-03

**Authors:** Yong Ma, Pengwei Qi, Jun Ma, Le Wei, Liang Zhao, Jian Cheng, Yanhui Su, Yuting Gu, Yuebin Lian, Yang Peng, Yanbin Shen, Liwei Chen, Zhao Deng, Zhongfan Liu

**Affiliations:** ^1^ Soochow Institute for Energy and Materials Innovations College of Energy Key Laboratory of Advanced Carbon Materials and Wearable Energy Technologies of Jiangsu Province Soochow University Suzhou 215006 China; ^2^ School of Chemistry and Chemical Engineering Shanghai Jiao Tong University Shanghai 200240 China; ^3^ i‐Lab CAS Center for Excellence in Nanoscience Suzhou Institute of Nano‐Tech and Nano‐Bionics (SINANO) Chinese Academy of Sciences Suzhou 215123 China; ^4^ Center for Nanochemistry (CNC) Beijing Science and Engineering Center for Nanocarbons College of Chemistry and Molecular Engineering Peking University Beijing 100871 China

**Keywords:** artificial solid/electrolyte interphase, chemical vapor depositio‐grown graphene, lithium–air batteries, water‐resistant anodes

## Abstract

One of the key challenges in achieving practical lithium–air battery is the poor moisture tolerance of the lithium metal anode. Herein, guided by theoretical modeling, an effective tactic for realizing water‐resistant Li anode by implementing a wax‐assisted transfer protocol is reported to passivate the Li surface with an inert high‐quality chemical vapor deposition (CVD) graphene layer. This electrically conductive and mechanically robust graphene coating enables serving as an artificial solid/electrolyte interphase (SEI), guiding homogeneous Li plating/stripping, suppressing dendrite and “dead” Li formation, as well as passivating the Li surface from moisture erosion and side reactions. Consequently, lithium–air batteries fabricated with the passivated Li anodes demonstrate a superb cycling performance up to 2300 h (230 cycles at 1000 mAh g^−1^, 200 mA g^−1^). More strikingly, the anode recycled thereafter can be recoupled with a fresh cathode to continuously run for 400 extended hours. Comprehensive time‐lapse and ex situ microscopic and spectroscopic investigations are further carried out for elucidating the fundamentals behind the extraordinary air and electrochemical stability.

## Graphical abstract

Water‐resistant Li anodes are developed by implementing a wax‐assisted transfer protocol to passivate the Li surface with high‐quality CVD graphene. Serving as an artificial solid/electrolyte interphase, the hydrophobic, conductive, and robust graphene coating can effectively dissipate local surface charges, homogenize Li deposition, suppress dendrite growth, and protect the Li surface from parasitic reactions with organic electrolytes and moisture.

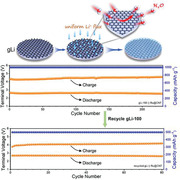

## 1 Introduction

Rechargeable Li–air battery is one of the most tempting electrochemical energy storage solutions due to its ultrahigh theoretic energy density (3500 Wh kg^−1^), tenfold higher than that of the state‐of‐the‐art Li‐ion battery today, and the elimination of reactant tanks as those required by fuel cells and flow batteries.^[^ ^1^ ^]^ However, its prevailing applications outside the laboratory is still hindered by critical technical challenges including the low energy efficiency caused by large discharge/charge overpotentials,^[^ ^2^ ^]^ the shortened lifetime due to electrolyte decomposition,^[^ ^3^ ^]^ and the corrosion and safety issues related to the use of Li metal anodes.^[^ ^4^ ^]^ Each of these issues needs to be earnestly taken care of and addressed in a systemic fashion before the technology can be ultimately deployed to benefit human life.^[^ ^5^ ^]^ In the past decade, remarkable advances have been gained in improving the catalytic potency of the air cathode catalyst,^[^ ^6^ ^]^ promoting the stability and redox‐mediating ability of the electrolyte,^[^ ^7^ ^]^ and comparting the cell configuration for enhanced performance.^[^ ^8^ ^]^ However, not much attention has been specifically paid to the anode side of Li–air batteries with respect to the air and moisture stability, which are crucial for extending the cycling life and improving the operational safety in ambient environment.^[^ ^9^ ^]^

The challenge in dealing with lithium anodes lies in the intrinsic high chemical reactivity of Li, the heterogeneous and unstable solid/electrolyte interphase (SEI), and the cyclic volume expansion caused by accumulative deposition of Li dendrite and “dead” Li.^[^ ^10^ ^]^ Numerous efforts have been devoted to stabilize the lithium anodes, including the modification of electrolyte with SEI‐stabilizing additives,^[^ ^11^ ^]^ construction of artificial SEI and protection layers,^[^ ^12^ ^]^ host of Li metal in conductive and lithiophilic 3D scaffolds,^[^ ^13^ ^]^ and adoption of solid inorganic/polymer electrolyte or spacers.^[^ ^14^ ^]^ Among the various tactics, the passivation of lithium metal surface by 2D materials has attracted particular attention by efficiently mediating Li plating/stripping and suppressing dendrite formation.^[^ ^15^ ^]^ For instance, Langmuir‐Blodgett artificial SEI was constructed with phosphate‐functionalized reduced graphene oxides (rGO) to achieve stable operation of Li–nickel cobalt manganese oxide batteries with minimized n/p ratio.^[^ ^15b^ ^]^ 2D MoS_2_ was directly sputtered onto Li metal serving as a protective layer, and greatly improved the performance of Li–S batteries.^[^ ^15c^ ^]^ Through solvent evaporation‐assisted self‐assembly, our group successfully passivated the Li anodes with a mosaic rGO layer, leading to superb cycling performance of Li–sulfurized polyacrylonitrile cells.^[^ ^15d^ ^]^ Nonetheless, despite the great progress achieved by implementing these 2D protection layers, which are mostly hydrophilic and defective, there have been no demonstration of water/moisture‐resistance, which is highly desired by applications such as Li–air battery with an open‐cell configuration, as a tiny amount of H_2_O could result in severe corrosion of the Li anode leading to significant performance deterioration.^[^ ^16^ ^]^

Herein, guided by physical modeling, we report a facile but efficacious method for realizing water‐resistant Li anodes by implementing a wax‐assisted transfer protocol to passivate the Li surface with high‐quality CVD graphene films. Serving as an artificial SEI, the conductive and robust graphene coating can effectively dissipate local surface charges, homogenize Li deposition, suppress dendrite growth, and protect the Li surface from parasitic reactions with organic electrolytes and moisture. As a result, high Coulombic efficiency of Li plating/stripping and long‐term cycling reversibility were witnessed in both half and symmetric cells. Li–air batteries fabricated with the protected Li metal anodes demonstrated an impressive long cycling of 2300 h, and even more strikingly, the recycled anode can be further recoupled with a fresh cathode and continuously operate for extended hours. To help understand the performance enhancement brought by the CVD graphene protection layer, a full spectrum of microscopic and spectroscopic techniques was exploited for time‐lapse and ex situ investigations.

## 2 Results and Discussions

### 2.1 Simulation of Li^+^ Flux on Bare Lithium and Graphene‐Coated Lithium

**Figure 1:**
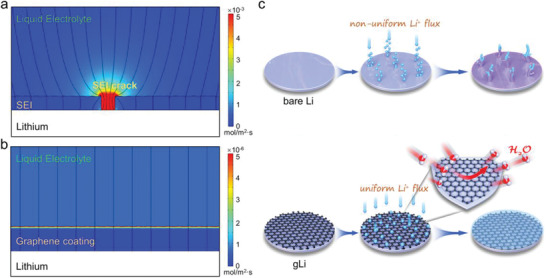
a,b) Simulations of Li^+^ flux and distribution on the bare Li and graphene‐coated Li (gLi), respectively. c) The schematic diagram showing the graphene protection layer against dendrite formation and moisture attack.

First, the interfacial Li^+^ flux and distribution on bare Li with and without graphene coating was modeled using the COMSOL Multiphysics toolbox. For bare Li, a layer of conventional SEI with a pinhole was constructed on the Li surface, whereas on the graphene‐coated Li (gLi), the graphene coating serves as a coherent artificial SEI. The simulation of the Li^+^ flux was based on three key parameters of the electrolyte and SEI, including the ionic conductance, diffusion constant, and surface roughness. Table S1, Supporting Information, list the values of Li^+^ ionic conductance and diffusion constant in the electrolyte, SEI, and graphene, according to previous reported values. The roughness factors of the SEI and graphene are set to 2 and 0.5, respectively. As illustrated in **Figure** **1a**, the pinhole induced by SEI rupture during repeated plating and stripping creates a hot spot of Li^+^ flux on the bare Li surface, leading to localized Li deposition that would eventually cause dendrite growth and “dead Li” formation (Figure 1c). In contrast, on the gLi surface the Li^+^ flux is uniform due to the coherent graphene film and its high charge conductance, and generates a thin zone of enhanced Li^+^ concentration at the electrolyte/graphene interface owing to the difference in Li^+^ diffusion constant between the two phases (Figure 1b). Accordingly, the Li^+^ flux inside the graphene coating, albeit still uniform, is lower than that in the electrolyte. Based on the simulation here, it can be then postulated that a robust and conductive graphene coating should be in favor of homogeneous Li^+^ deposition, both on and across the graphene layer, and thereby stabilize the Li anode. To validate the modeling results, we'll next seek after experimental evidences by passivating the Li anode surface with high‐quality CVD graphene.

### 2.2 Fabrication and Characterization of Graphene‐Coated Lithium Anodes

**Figure 2:**
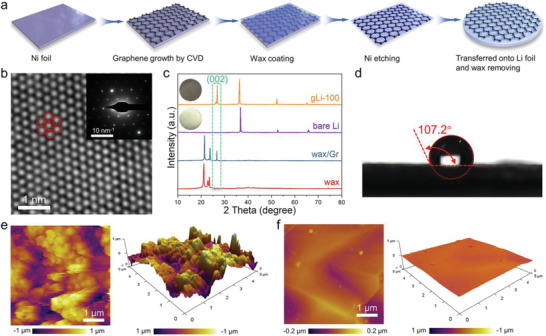
Fabrication and characterization of the gLi anodes. a) The schematic diagram showing the coating of Li surface with a CVD graphene film via the wax‐assisted protocol. b) FFT‐filtered aberration corrected TEM image taken on the CVD graphene film. Insert is the corresponding selected‐area electron diffraction (SAED) pattern. c) XRD pattern of the gLi‐100 anode displaying the co‐existence of Li and graphene signatures. Inserts are the optical images of the corresponding bare‐Li and gLi‐100 anodes. d) Measurement of water contract angle on the gLi‐100 electrode. AFM top‐view and 3D topograph of the e) bare‐Li and f) gLi‐100 anodes.

**Figure** **2a** shows the flowchart for coating both sides of the Li foil with the chemical vapor deposition (CVD)‐grown graphene film via a wax‐assisted protocol, which was previously developed in our group as a facile alternate for the classic PMMA method for graphene transfer.^[^ ^17^ ^]^ The notable advantages of our wax method lie in the facilitated sample handling and template removal owing to the great thermal property and solubility of paraffin. More importantly, the transferred high‐quality graphene film with excellent integrity can maintain seamless and conformal contact with the underlying Li to avoid perforation and delamination. Specifications of the graphene coating such as thickness can be readily varied by tuning the CVD growth parameters (Figure S1, Supporting Information). For instance, by adjusting the H_2_/CH_4_ ratio and the growth time, graphene thin films of ≈200, 100, and 30 nm were obtained (Figure S2, Supporting Information), and correspondingly the coated Li foils are denoted as gLi‐200, gLi‐100, and gLi‐30. Of note, the gLi‐100 sample manifested the lowest electrochemical impedance in symmetric cells among all gLi‐*x* and bare‐Li samples (Figure S3, Supporting Information), possibly owing to a trade‐off between the beneficial surface passivation and escalated Li^+^ diffusion resistance (as suggested by the above modeling). Thus, all the following characterizations and electrochemical assessments will be based on gLi‐100.

First of all, Raman spectrum taken on the as‐grown graphene thin film shows a broad 2D peak and the absence of the defect‐associated D band (Figure S4, Supporting Information), indicating defect‐free and high crystalline nature of the sample, which is further affirmed by the atomic image and electron diffraction pattern (Figure 2b) acquired using high‐resolution transmission electron microscopy (HR‐TEM). As shown in Figure 2c, the XRD pattern of gLi‐100 comprises both signatures from the graphene coating and the underlying Li metal, with the distinct peak at 2*θ* = 26° corresponding to the (002) planes of multilayer graphene. Furthermore, scanning electron microscope (SEM) images (Figure S5, Supporting Information) of the as‐obtained gLi‐100 show clearly grain boundaries of graphene, further confirming the successful transfer of graphene film onto the Li metal surface. Atomic force microscopy (AFM) operated under the peak‐force tapping mode measured an average Young's modulus of 31.2 ± 4.3 GPa for gLi‐100 (Figure S6 and Table S2, Supporting Information), far exceeding that of 4.9 GPa reported for Li metal,^[^ ^18^ ^]^ and that of 0.15 GPa reported for a conventional SEI,^[^ ^19^ ^]^ which should help mechanically stabilize the Li surface. The surface of gLi‐100 further shows a water contact angle of 107.2° (Figure 2d), similar to that of the pristine graphene film (105.9°, Figure S7, Supporting Information). Collectively, the above observations clearly show that the CVD graphene thin film has been successfully coated onto the Li foil and its high hydrophobicity and mechanical strength should help protect the underneath lithium surface. What's more, the wax‐transferred graphene coating on Li foil can be easily scaled up due to the facile fabrication process, which is demonstrated by the large 95 mm × 25 mm gLi‐100 foil shown in Figure S8, Supporting Information. We believe that further engineering efforts should help continuously scale up the gLi fabrication, ultimately making the innovation commercially feasible.

The surface morphology of gLi‐100 versus bare Li was further investigated by AFM installed in a glove box. Strikingly, gLi‐100 shows a much smoother topograph than the bare Li does (Figure 2e,f). A few surface ridges, which are characteristic of the CVD‐grown graphene film, can be clearly seen on gLi‐100, while the bare Li presents highly rugged morphology exhibiting many surface pits and protrusions. This difference in surface roughness observed by AFM further validates our physical models with varied roughness factors (Figure 1a,b). As suggested by the modeling results, when used for Li metal anodes the conductive and smooth surface of gLi‐100 should help homogenize local surface charge density and evenly dissipate Li^+^ flux by avoiding the “tip effect” (Figure 1c).^[^ ^20^ ^]^

### 2.3 Air and Electrochemical Stability

**Figure 3:**
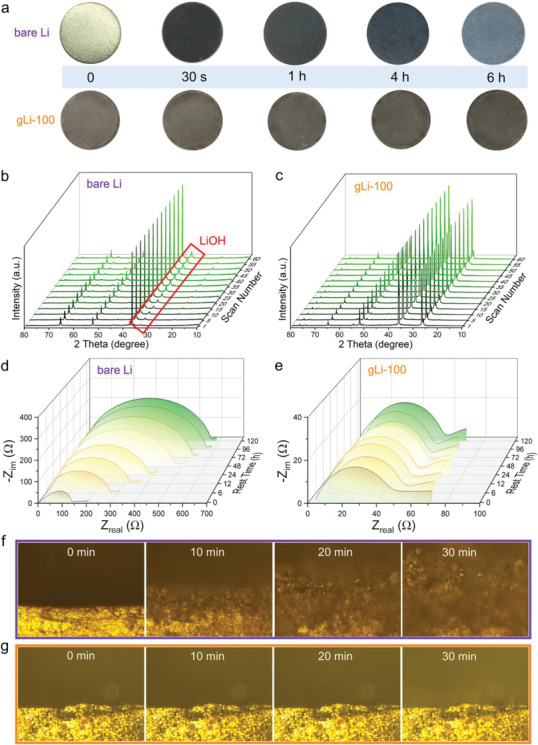
Air and electrochemical stability of gLi‐100 versus bare Li. a) Serial optical images of the bare‐Li and gLi‐100 anodes exposed to air for 6 h. Time‐lapse XRD patterns of b) bare Li and c) gLi‐100 exposed to air. Time‐lapse EIS of d) bare Li and e) gLi‐100 in symmetric cells measured at the open‐circuit potential after resting for various periods. Time‐lapse optical microscopy showing the Li plating process on the f) bare‐Li and g) gLi‐100 electrodes.

The air stability of the as‐prepared gLi‐100 anodes was examined by both the time‐lapse photography and XRD. When exposed to ambient air at room temperature with a relative humidity of 45–60%, the bare Li turned into rusty and dark appearance immediately upon taking out from the glove box (**Figure** **3a**). After 6 h, the color of the bare Li changed to bluish grey, indicative of severe surface corrosion. By contrast, the surface of gLi‐100 maintained an overall unchanged appearance and texture after the same period of exposure, corroborating the great protection of the graphene coating against air moisture. More impressively, the gLi‐100 anode can be even tossed into water without causing any notable reaction (Video S1, Supporting Information), whereas the bare Li flares up immediately (Video S2, Supporting Information). The time‐lapse XRD further shows the gradual emergence of a LiOH peak at 2θ = 33.1° on bare Li (Figure 3b) during the 6 h testing period in air (humidity of the XRD chamber was controlled at ≈50%), while no such peak was observed for gLi‐100 (Figure 3c). The observations made here, in conjunction with previous water contact angle measurements, endorse the superb air and moisture stability of gLi‐100.

Next, the electrochemical stability of gLi‐100 was examined with respect to its reactivity with electrolyte and suppression of dendrite growth. For that, symmetric cells of gLi‐100 were assembled for time‐lapse electrochemical impedance spectroscopy (EIS) and optical microscopy measurements, and the results are compared to those obtained for bare Li. At the rest state under open‐circuit potential, the impedance of the bare Li symmetric cell increased from 140 Ω at 0 h to 570 Ω after 120 h (Figure 3d), which can be ascribed to severe SEI formation due to the spontaneous reaction between Li and electrolyte. In stark contrast, the symmetric gLi‐100 cell displays fairly consistent Nyquist plots with a stabilized *R*_SEI_ at around 50 Ω during the entire 120 h testing period (Figure 3e). These observations strongly support that the highly crystalline graphene film can effectively passivate the Li surface to suppress parasitic side reactions with the organic electrolyte.

In situ time‐lapse optical microscopy was carried out to monitor the Li plating behavior on the gLi‐100 and bare‐Li surface (Figure 3f,g). Strikingly, while in no time the bare Li surface evolved into a highly chaotic and mossy morphology, indicative of severe dendrite and “dead” Li formation, the gLi‐100 electrode maintained both clean surface and cross section without obvious dendrite formation during the entire 30 min plating period. This observation is further coincided with the smooth AFM topograph after plating 5 mAh cm^−2^ of Li onto the gLi‐100 surface (Figure S9, Supporting Information), whereas the bare‐Li surface after plated with the same amount of Li was too rough to be imaged by AFM. Taken together from the above time‐lapse microscopic and spectroscopic studies, we can now conclude that the graphene coating on Li anodes endows not only air and chemical stability, but also helps guide smooth Li deposition with alleviated dendrite growth, just as predicted by previous modeling results.

### 2.4 Electrochemical Properties of Half‐ and Symmetric‐Cells

**Figure 4:**
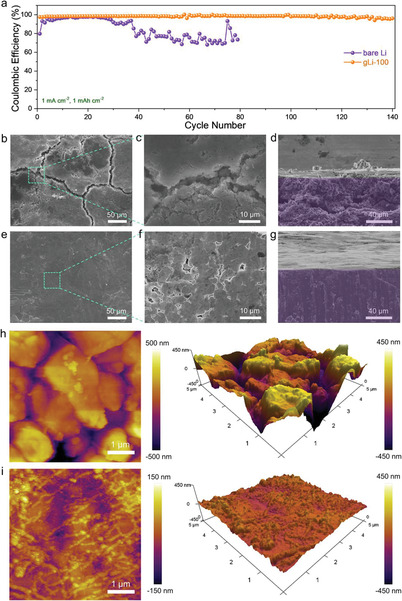
Properties and characterizations of gLi‐100||Cu half‐cells. a) Comparison of Coulombic efficiency in half‐cells for gLi‐100 and bare Li. b,c) SEM top‐view of different magnification and d) cross‐sectional images of the disassembled bare‐Li electrode after 100 cycles. e,f) SEM top‐view of different magnification and g) cross‐sectional images of the disassembled gLi‐100 electrode after 100 cycles. AFM top‐view and 3D topograph of the disassembled h) bare‐Li and i) gLi‐100 electrodes after 100 cycles.

To inspect the Coulombic efficiency of Li plating/stripping, half‐cells with gLi‐100 or bare Li serving as the working electrode and Cu foil serving as the counter electrode were assembled. At a current density of 1 mA cm^−2^, the gLi‐100‖Cu half‐cell was able to maintain a Coulombic efficiency above 97% for over 140 cycles, whereas the control Li‖Cu half‐cell showed a much inferior cycling stability, exhibiting chaotic Coulombic efficiency fluctuation after only 30 cycles (**Figure** **4a**), which is typically associated with the cyclic SEI fracture and repetitive formation/dissolution of dendrite and “dead” Li.^[^ ^21^ ^]^ A closer examination on the serial plating/stripping curves revealed not only a more reversible cyclic capacity on gLi‐100 (Figure S10, Supporting Information), but also much reduced and stabilized charge/discharge hysteresis (Figure S11, Supporting Information). This reduced electrode polarization strongly evidences the facilitated Li plating/stripping across stabilized SEI enabled by the graphene protection layer.

Upon disassembling the half‐cells after 100 cycles, SEM images were taken to compare the bare Li and gLi‐100 surfaces. In the top‐view images of bare Li, extensive cracks can be observed on the roughened surface (Figure 4b). Inside the cracks, loose Li structures are clearly visualized (Figure 4c), indicative of excessive “dead” Li, which can be further witnessed from the cross‐sectional view showing delaminated and loosely packed morphology (Figure 4d). In stark contrast, the disassembled gLi‐100 electrode exhibits a smoother surface comprising island‐like Li domains (Figure 4e,f), apart from a dense and intact cross section (Figure 4g). Ex situ AFM was further exploited to reveal the evolution of surface topograph and texture in greater details for both the bare‐Li and gLi‐100 electrodes. After cycling the Li‖Cu half‐cell for 50 cycles, the local morphology of the bare‐Li electrode appears to be smoother when compared to the pristine Li surface (Figure S12a, Supporting Information, vs Figure 2e). This is possibly due to the formation of SEI that effectively flattens local Li topograph by filling big surface pits. Nonetheless, its surface roughness is still significantly larger when compared to that of gLi‐100 after 50 cycles, exhibiting highly grained texture (Figure S12b, Supporting Information). After 100 cycles, the granular protuberances on the bare Li electrode grew into loosely packed larger agglomerates (Figure 4h), echoing the above SEM observation. By contrast, the gLi‐100 electrode after 100 cycles still displays an overall flat and smooth surface exhibiting enormous small granules (Figure 4i). In general, our half‐cell study above clearly shows the more efficient Li plating/stripping on gLi‐100, thanks to the graphene coating facilitating homogeneous Li deposition and suppressing Li dendrite formation. In addition, the domain structure viewed from SEM and grain texture viewed by AFM suggest at least a portion of Li^+^ are deposited atop the graphene layer, which is consistent with previous simulation.

**Figure 5:**
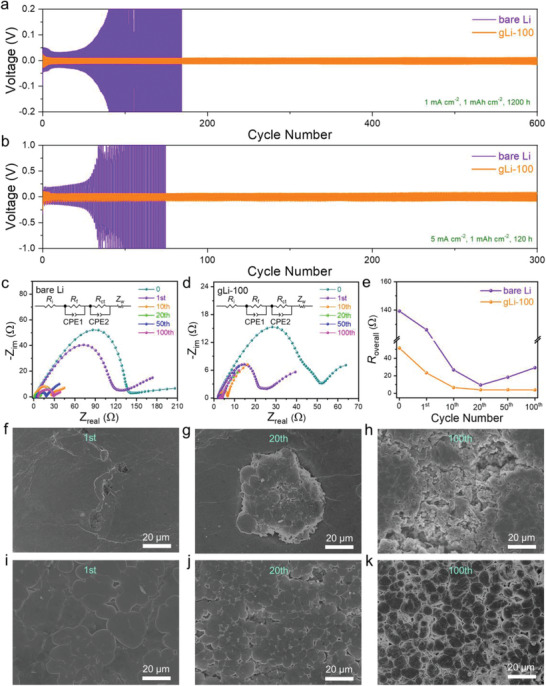
Electrochemical properties of symmetric cells. Galvanostatic cycling tests of symmetric bare‐Li and gLi‐100 cells at a) 1 mA cm^−2^ and b) 5 mA cm^−2^ with a fixed capacity of 1 mAh cm^−2^. EIS of the c) bare‐Li and d) gLi‐100 cells at different cycle status. Inserts are the corresponding equivalent circuit diagrams. e) Comparison of the overall impedance (*R*_overall_) from EIS in (c) and (d) for bare Li versus gLi‐100. Ex situ SEM images of the disassembled f–h) bare‐Li and i–k) gLi‐100 electrodes from symmetric cells at different cycle status.

Symmetric cells assembled with the bare‐Li or gLi‐100 electrodes were further cycled to interrogate the long‐term passivation effect of the CVD graphene layer. **Figure** **5a** presents the galvanostatic cycling profiles of both the bare‐Li and gLi‐100 symmetric cells under a fixed areal capacity of 1 mAh cm^−2^ at 1 mA cm^−2^. It can be seen that the gLi‐100 electrode shows a superb cycling stability with a charge/discharge overpotential as low as 10 mV for the entire 600 testing cycles (1200 h, Figure S13, Supporting Information), whereas the bare Li manifests a severe voltage hysteresis with the charge/discharge overpotentials rapidly surging to 200 mV after just 80 cycles (160 h). More impressively, even at a high current density of 5 mA cm^−2^, the gLi‐100 symmetrical cell could still deliver a long‐term stability for over 300 cycles (120 h) with a stabilized overpotential less than 80 mV (Figure 5b and Figure S14, Supporting Information), which is, again, far superior to the bare‐Li cell exhibiting a short‐lived unstable voltage profile. The greatly improved cycling performance of gLi‐100 with lowered voltage hysteresis in symmetrical cells can be reasonably attributed to the more conductive and robust graphene coating in substitution of the conventional SEI, effectively mitigating local charge accumulation, parasitic side reactions, as well as cyclic SEI fracture.

To consolidate the above point of view with regard to the electrode/electrolyte interfacial stability, EIS measurements were carried out to monitor the impedance evolution on symmetric cells at various cycling states (under 1 mAh cm^−2^ at 1 mA cm^−2^, Figure 5c,d). For the argument of simplicity and facilitation of comparison, the overall impedance *R*_overall_ (i.e., the sum of internal resistance [*R*_i_], interfacial resistance [*R*_f_], and charge‐transfer resistance [*R*_ct_]) is adopted for comparing the cell impedance as a whole, and can be directly read from the Nyquist plot at the end of the semi‐circle at low frequency. For both the bare‐Li and gLi‐100 electrodes, the values of *R*_overall_ dropped continuously in the first 20 cycles (from 139 to 10 Ω for bare Li and 52 to 4 Ω for gLi‐100, Figure 5e), indicating an initial activation and conditioning process.^[^ ^22^ ^]^ Afterward, *R*_overall_ of the bare Li symmetric cell increased again to 18 Ω at the 50th cycle and further to 39 Ω at the 100th cycle, as a result of the accumulation of SEI layer due to its repetitive rupture and regeneration during cycling, whereas *R*_overall_ for gLi‐100 kept mostly unchanged at ≈4 Ω for the rest of cycles (Figure 5e). These observed trends in impedance evolution are in good agreement with the evolution of voltage hysteresis in Figure 5a, both attesting to the greatly reduced and stabilized electrode polarization on gLi‐100 upon cycling, apart from the much‐improved charge transfer kinetics.^[^ ^23^ ^]^ Moreover, the slopes of the Z′–*ω* ^−1/2^ curves in Figure S15, Supporting Information, for gLi‐100 are generally smaller than those observed on the bare‐Li symmetrical cell, indicating improved Li^+^ diffusion in the artificial SEI.^[^ ^24^ ^]^

Similar to the observations on the gLi‐100‖Cu and Li‖Cu half‐cells, SEM images taken on the bare Li and gLi‐100 electrodes disassembled from the symmetric cells after 100 cycles reveal a cracked surface morphology of the former, and a homogeneous domain‐rich morphology of the latter (Figure S16, Supporting Information). The high‐resolution ex situ SEM images in Figures 5f–h and 5i–k clearly illustrate the morphological evolution of the bare‐Li and gLi‐100 electrodes, respectively, along the cycling process. The bare‐Li electrode after the 1st cycle of stripping and plating displays many surface pits (Figure 5f and Figure S16a, Supporting Information), serving as the preferential nucleation sites for subsequent non‐uniform Li deposition (Figure 5g and Figure S16b, Supporting Information). During the successive cycling, the rough surface regions due to uncontrolled Li deposition gradually expand and coalesce, and finally develop into surface cracks comprising excessive Li dendrites and “dead” Li (Figure 5h and Figure S16c, Supporting Information). On the other hand, the Li deposition on the gLi‐100 surface is more homogeneous, forming increasingly densely packed Li domains as the cycling goes on (Figure 5i–k and Figure S16d–f, Supporting Information). Once again, these microscopic observations are in good agreement with previous theoretic modeling, corroborating the better interfacial stability of gLi‐100, as well as its lowered and stabilized *R*_overall_ values upon cycling. X‐ray photoelectron spectroscopy (XPS) characterization was performed to analyze the composition of the SEI layer on both bare‐Li and gLi‐100 after cycling (Figure S17, Supporting Information). Compared with the C 1s spectrum of the bare‐Li electrode, that of gLi‐100 exhibits more prominent C─C species from graphene but significantly reduced peak intensities of COR, C═O, and HCO_2_Li/COOR, indicating mitigated electrolyte decomposition. In addition, a strong peak of C─F_3_ is observed for the bare‐Li electrode, ascribable to the decomposition of LiTFSI.^[^ ^25^ ^]^ When comparing the O 1s spectra, more oxygen contents were observed on the SEI of bare Li with an extra peak of Li_2_O at 528.3 eV, which should come from the decomposition of DOL.^[^ ^26^ ^]^ Furthermore, in the Li 1s spectra the intensity ratio of LiF on the surface of gLi‐100 electrode is notably smaller than that on the surface of bare‐Li, further affirming the suppressed electrolyte decomposition.^[^ ^27^ ^]^ Taken together, it is evident that the CVD‐grown graphene enables serving as an artificial SEI layer to stabilize the interface and mitigate side reactions.

### 2.5 Demonstration of Lithium–Air Batteries

**Figure 6:**
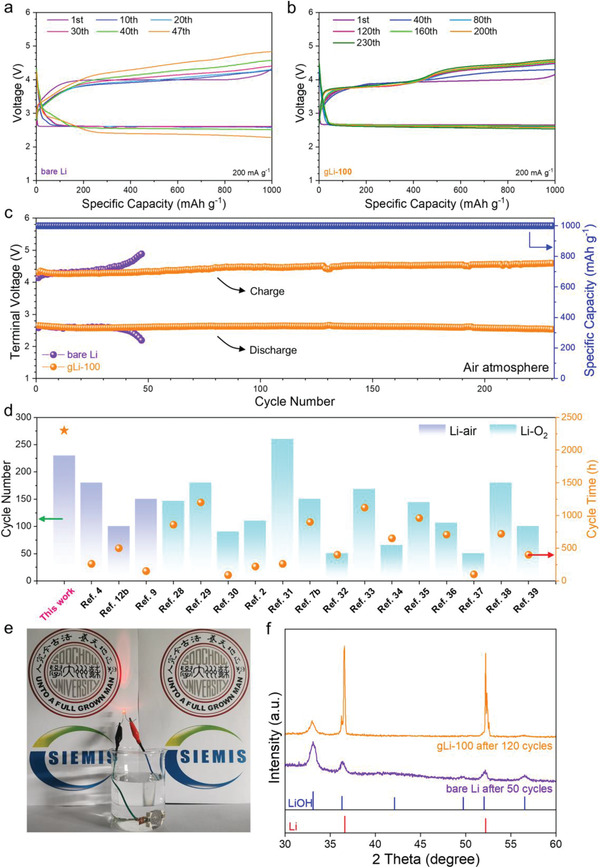
Demonstration of long‐cycle and moisture‐stable Li–air batteries. Serial discharge/charge profiles of a) bare‐Li||Ru@CNT and b) gLi‐100||Ru@CNT cells cycled at 200 mA g^−1^ with a cut‐off capacity of 1000 mAh g^−1^. c) Long‐term cycling performances of the bare‐Li||Ru@CNT and gLi‐100||Ru@CNT cells. d) Comparison of the cycling performance with previous reports on Li–air batteries and Li–O_2_ batteries. e) Lighting an LED bulb with the gLi‐100||Ru@CNT cell immersed in water. f) XRD patterns taken on the disassembled bare‐Li and gLi‐100 electrodes after cycling for 50 and 120 cycles, respectively.

Encouraged by the remarkable air and electrochemical stability of gLi‐100, Li–air batteries were fabricated using gLi‐100 as the anode and ruthenium‐doped carbon nanotubes (Ru@CNT) as the classic cathode catalyst. The batteries were tested under a fixed capacity of 1000 mAh g^−1^ at the current density of 200 mA g^−1^ between 2.2 and 4.6 V (vs Li/Li^+^) in air, with the bare Li serving for control studies. To ensure the testing reproducibility, cylinder compressed air was used with a fixed water content of ≈5600 ppm as verified by mass spectroscopy (Figure S18, Supporting Information). **Figures** **6a** and 6b display the serial discharge/charge profiles at various cycling states for Li–air batteries comprising the bare Li and gLi‐100 anodes, respectively. For the bare‐Li‖Ru@CNT cell, both the discharging and charging profiles deteriorate gradually with increasing cycles. The charging terminal voltage surged to 4.8 V after just 47 cycles, which is considered highly detrimental to the electrolyte stability. By contrast, the gLi‐100‖Ru@CNT cell was able to operate steadily over 230 cycles (2300 h) with the charge/discharge curves mostly overlapped. Of note, the cycling stability of the gLi‐100 cell is more than five times better than that of the bare‐Li cell when compared at the same cut‐off terminal voltage (Figure 6c). Apparently, the graphene protection layer enables greatly extending the anode lifetime in Li–air batteries by synergistically improving the Coulombic efficiency of Li plating/stripping, suppressing dendrite and “dead” Li formation, and passivating the Li surface from moisture attack.

More impressively, when the gLi‐100 anode was disassembled from the Li–air battery after running for 230 cycles and recoupled with a fresh Ru@CNT cathode, the new cell can continuously run for another 80 cycles at a fixed cycling capacity of 500 mAh g^−1^ under the cutting‐off terminal voltage of 4.2 V (Figure S19, Supporting Information). This experiment strongly suggests that the increased overpotential in the first cycling trial was mainly due to the deactivation of the Ru@CNT cathode, and/or the evaporation and decomposition of electrolyte after prolonged cycling. Apart from the cycling stability, the gLi‐100‖Ru@CNT cell also demonstrated superior rate capability when the current density was ramped up from 100 to 1000 mA g^−1^ and then back to 100 mA g^−1^, whereas the bare‐Li cell failed at 500 mA g^−1^ (Figure S20, Supporting Information). In virtue of the great passivation effect from the graphene coating, the cycling performance of the gLi‐100‖Ru@CNT cell demonstrated here is ranked among the best Li–air batteries reported today, and is even superior to many of the state‐of‐the‐art Li–O_2_ batteries tested in pure oxygen (Figure 6d).^[^ ^2^, ^4^, ^7^, ^9^, ^12^, ^28^, ^29^, ^30^, ^31^, ^32^, ^33^, ^34^, ^35^, ^36^, ^37^, ^38^ ^]^ More absurdly here, to showcase the superb water tolerance of the gLi‐100 cell in operation, we immersed the as‐fabricated Li–air battery in water and found it can still light up an LED for a short period of time with the preperfused air (Figure 6e and Video S3, Supporting Information).

Last, post‐mortem and operando characterizations employing SEM, XRD and in situ differential electrochemical mass spectroscopy (DEMS) were carried out to seek insights into the performance enhancement brought by the graphene protection layer. As expected, the bare‐Li anode disassembled from the Li–air battery after 50 cycles revealed a significantly eroded surface full of gravelly “dead” Li (Figure S21a,b, Supporting Information), whereas the gLi‐100 anode retained a relatively smooth and compact surface even after 120 cycles (Figure S21c,d, Supporting Information). The corresponding XRD patterns in Figure 6f revealed that on the bare Li anode the intensity of LiOH peaks overwhelms that of the Li metal, while on gLi‐100 the peaks of metallic Li are still the prominent feature, corroborating the greatly inhibited moisture erosion and side reactions. This argument is further supported by in situ DEMS measurements, showing less CO_2_ elution from the charging process of the gLi‐100 cell when compared to that from the bare‐Li cell (Figure S22, Supporting Information). The higher CO_2_ elution from the latter is ascribed to the aggravated electrolyte decomposition. Furthermore, by quantifying the O_2_ evolution versus charge consumption during the charging process, the numbers of electron transfer per O_2_ molecule were determined to be 2.34 and 2.12 for the bare Li and gLi‐100, respectively, further attesting to the superior Faradaic efficiency of the latter with suppressed side reactions.

## 3 Conclusion

In summary, a physical model was first constructed to enlighten the more homogeneous Li^+^ flux atop the gLi surface versus bare Li. Then, for experimental validation, a wax‐assisted transfer method was implemented to coat Li metal anodes with the high‐quality CVD‐grown graphene films of various thicknesses. The thus fabricated and optimized gLi‐100 anodes demonstrate superb air and electrochemical stability, as evidenced by time‐lapse spectroscopic and microscopic studies, electrochemical and morphological characterizations on half‐ and symmetric‐cells, as well as the demonstration of stable Li–air batteries. Strikingly, after an impressive long cycling for 2300 h, the recycled gLi‐100 anode can be further recoupled with a fresh cathode and continuously run for extended hours. What's more, the Li anode was protected so well by the CVD graphene layer that it is water‐resistant. While relieved from the worry of Li‐water contact, the as‐fabricated Li–air battery can be even immersed into water and still operatable shortly with preperfused air. By constructing a conductive and inert graphene layer to guide homogeneous Li plating/stripping, suppress dendrite and “dead” Li formation, and passivate the Li surface from moisture erosion and side reactions, our work offers a practical solution for protecting the Li anodes in Li–air batteries to afford extraordinary electrochemical performances.

## Conflict of Interest

The authors declare no conflict of interest.

## Supporting information

Supporting Information

Supplemental Video 1

Supplemental Video 2

Supplemental Video 3

## Data Availability

The data that supports the findings of this study are available in the Supporting Information of this article.
