# Supplementary material for: Wax‐Transferred Hydrophobic CVD Graphene Enables Water‐Resistant and Dendrite‐Free Lithium Anode toward Long Cycle Li–Air Battery
Source: Adv Sci (Weinh). 2021 Jun 3;8(16):2100488. doi: 10.1002/advs.202100488 (PMC8373161; doi:10.1002/advs.202100488)
Supplement: Supplementary file 1 — Supporting Information [file ADVS-8-2100488-s001.pdf]

© 2021 Wiley-VCH GmbH

ADVANCED SCIENCE
Open Access

Supporting Information

for *Adv. Sci.,* DOI: 10.1002/advs.202100488

Wax-Transferred Hydrophobic CVD Graphene Enables Water-Resistant and Dendrite-Free Lithium Anode toward Long Cycle Li-Air Battery

Yong Ma, Pengwei Qi, Jun Ma, Le Wei, Liang Zhao, Jian Cheng, Yanhui Su, Yuting Gu, Yuebin Lian, Yang Peng, Yanbin Shen, Liwei Chen, Zhao Deng*, Zhongfan Liu

Supporting Information

Wax-Transferred Hydrophobic CVD Graphene Enables Water-Resistant and Dendrite-Free Lithium Anode toward Long Cycle Li-Air Battery

Yong Ma, Pengwei Qi, Jun Ma, Le Wei, Liang Zhao, Jian Cheng, Yanhui Su, Yuting Gu, Yuebin Lian, Yang Peng, Yanbin Shen, Liwei Chen, Zhao Deng*, Zhongfan Liu

Experimental Section:

1. Chemicals and Materials

Nickel foil of 25 μm thick (Ni foil, ≥99%) was purchased from Alfa Aesar. Paraffin wax, toluene (C_7H_8, ≥99.5%), hydrochloric acid (HCl, 36.0~38.0%) and ethanol (C_2H_6O, ≥99.5%) were provided by Sinopharm Chemical Reagent Co., Ltd. Hydrogen peroxide (H_2O_2) (80%) was purchased from Lingfeng Chemical Reagent Co., Ltd. Milli-Q water (>18.0 MΩ cm) was purified with a Sartorius arium mini ultrapure water system. Lithium disks of 15 mm in diameter (1 mm thick) and lithium foil (0.3 mm thick) were received from China Energy Lithium Co., Ltd.

2. CVD Growth of thin graphene films

graphene films were synthesized by CVD according to our previously reported work.[1] The growth parameters are illustrated in the schematic diagram shown in Supplementary Figure 1. In a typically experiment, Ni foils were first cut into 18 × 18

mm^2 and placed on the quartz substrate in a low-pressure CVD furnace. The quartz tube was evacuated by a vacuum pump and then the pressure was brought back to ~400 Pa by purging H_2. After that the furnace was heated to 1020 °C in 50 min under 50 sccm H_2. After an annealing period of 30 min, CH_4 was introduced into the furnace. Graphene films with different thickness were obtained by adjusting the flow rate of CH_4 and the growth time. Finally, the furnace was naturally cooled to room temperature before taking out the samples.

3. Fabrication of gLi anodes

The as-grown graphene film was transferred onto a lithium disk by a wax-assisted transfer method. Firstly, molten wax was drop cast onto the as-prepared nickel-graphene film (Ni-GF) sample heated at 90 °C. After cooling down to room temperature, the sandwich Ni-GF-Wax samples were immersed in 1 M HCl solution containing 1% H_2O_2 for 24 h to completely etch away the Ni substrate. Subsequently, the remained GF-Wax samples were gently rinsed by deionized water and ethanol for several times to wash away any residual etchant solution. After dried in a nitrogen flow, the GF-Wax samples were transferred into glove box. To prepare the gLi anode, a GF-Wax slice was placed onto the lithium disk/foil on top of a glass slide. Then, the glass slide was heated to 120 °C for 1 min to soften the wax for a better adherence of the graphene film to the underlying lithium surface. Afterwards, the whole Li/GF/Wax ensemble was immersed into toluene to remove the liquefied wax. To further completely remove any residual wax, the Li/GF disk was rinsed by hot fresh toluene (90 °C) for several times. With the evaporation of toluene, the graphene layer can then tightly adhere to the lithium substrate. After further dried under vacuum for 12 h, the gLi anode was obtained and stored inside the glove box for further use.

4. Materials characterization

Raman spectra were collected by a Horiba Jobin Yvon HR Evolution spectrometer with a laser excitation wavelength of 633 nm. The lattice structure of the obtained graphite film was examined by Spherical Aberration Corrected Transmission Electron Microscope (AC-TEM, FEI Titan Themis Cubed G2 300). The surface morphology was characterized by field-emission scanning electron microscopy (FE-SEM, Hitachi SU8010) and atomic force microscopy (AFM, Asylum Research Cypher ES, tapping mode). The crystalline structure was analyzed by powder X-ray diffraction (XRD, Bruker D8 Advance diffractometer equipped with Cu-Ka radiation source). The chemical composition of the SEI layer was investigated by X-ray photoelectron spectroscopy (XPS, ESCALAB 250Xi system by Thermal Fisher).

5. Electrochemical measurements

All cells were assembled in an Ar-filled glove box with O_2 and H_2O contents below 0.1 ppm. For half and symmetric cells, the electrolyte composed of 1 M Lithium bis(trifluoromethylsulphonyl)imide (LiTFSI) in 1,3-dioxolan (DOL)/1,2-dimethoxyethan (DME) (v : v = 1 : 1) with 1% Lithium nitrate ($LiNO_3$) and the Celgard PP separator were used. Li-air batteries were assembled using punctured CR2032-type coin cells. The classic Ru@CNT air cathodes were prepared as follow. 100 mg commercial CNTs and 23.1 mg $RuCl_3\cdot xH_2O$ were first dispersed in 60 mL distilled water under vigorous stirring. The resulted dispersion was then transferred to a 100 mL Teflon-lined stainless-steel autoclave and hydrothermally treated at 120 °C for 6 h. After harvesting the precipitates by centrifugation, the obtained mixture was heated at 600 ºC for 3 h in a tube furnace under Ar atmosphere. The as-obtained Ru@CNTs catalyst and the polyvinylidene fluoride (PVDF) binder (80 : 20 in mass percentage) were mixed in N-Methyl-2-pyrrolidone (NMP) and then uniformly sprayed onto the carbon paper with a mass loading of 1.5 mg cm^{-2}. A glass

fiber separator (Whatman GF/D) soaked with the electrolyte containing 1 M Lithium bis(trifluoromethylsulphonyl)imide in triethylene glycol dimethyl ether (LiTFSI/TEGDME) was employed to separate the cathode and anode. The assembled Li-air batteries were evaluated in a battery tester filled with cylinder compressed air and rested for 5 h before testing. Galvanostatic cycling tests were conducted by the Land CT 2001A system at room temperature. Electrochemical impedance spectroscopy (EIS) was measured with an AC voltage amplitude of 5 mV from 100 kHz to 0.01 Hz on the Zennium E4 electrochemical workstation.

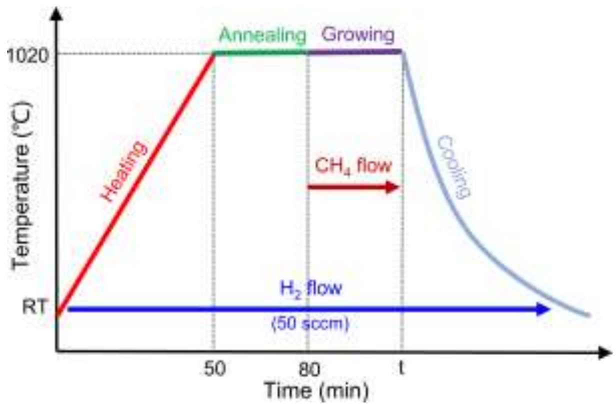

Figure S1. Schematic illustration of the CVD process for growing graphene thin films on Ni.

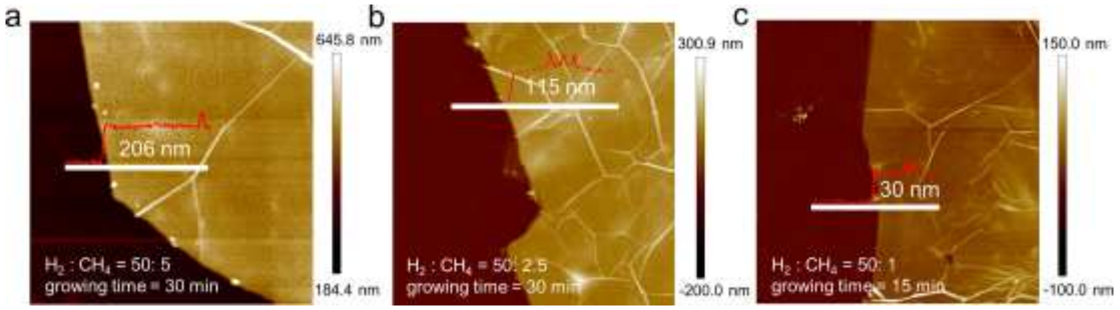

Figure S2. a-c) AFM images taken on the CVD graphene films of various thickness.

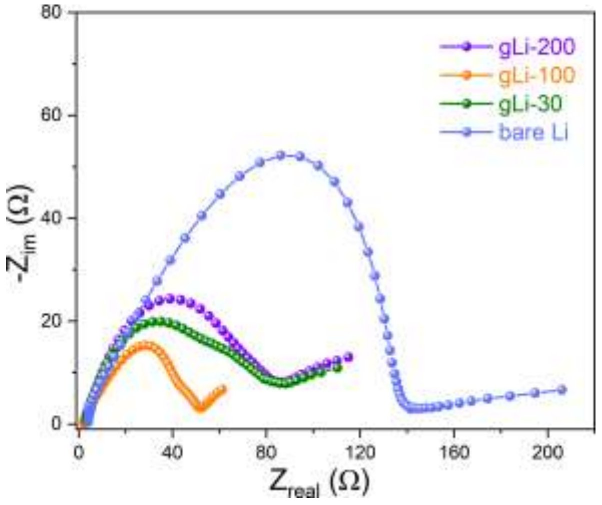

Figure S3. Electrochemical impedance spectra of the bare Li and gLi-*x* measured in symmetric cells at open-circuit potential.

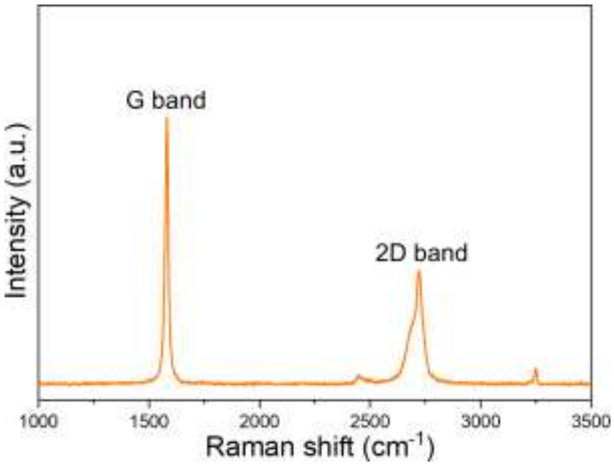

Figure S4. Raman spectra taken on the CVD graphene film.

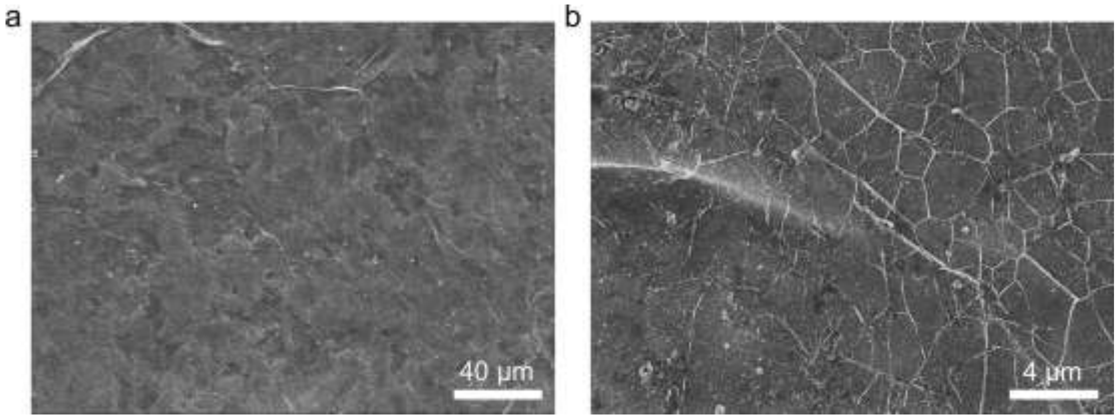

Figure S5. SEM images of the as-obtained gLi-100.

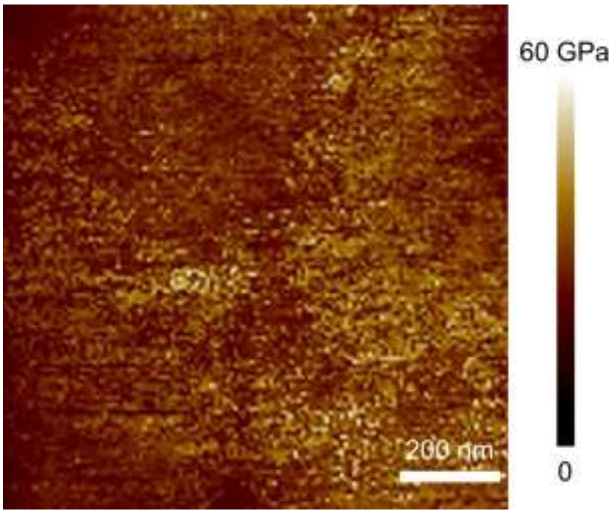

Figure S6. Mapping of Young’s modulus on gLi-100 under AFM peak-force mode.

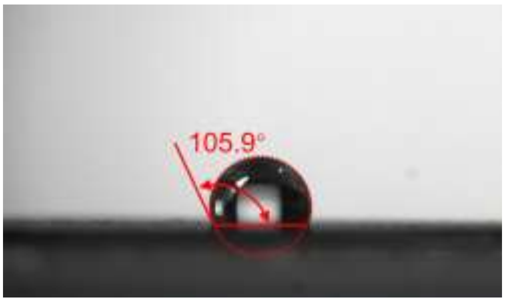

Figure S7. Measurement of water contract angle on the as-prepared CVD graphene film.

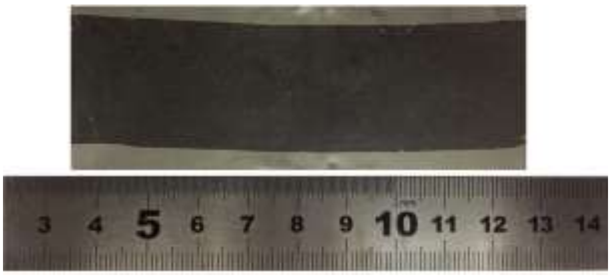

Figure S8. The photograph of a large gLi-100 foil of 95 mm ×25 mm.

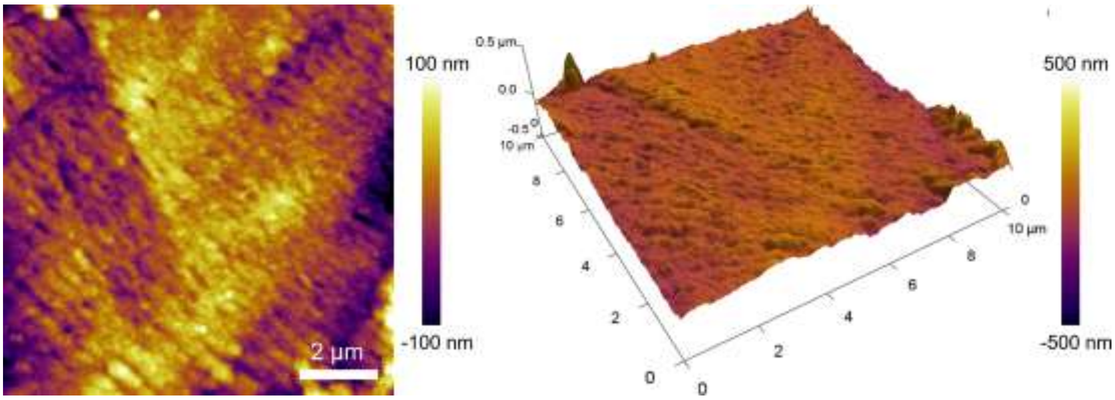

Figure S9. AFM top-view and 3D topograph of the gLi-100 electrode after plating 5 mAh cm^{-2} of Li.

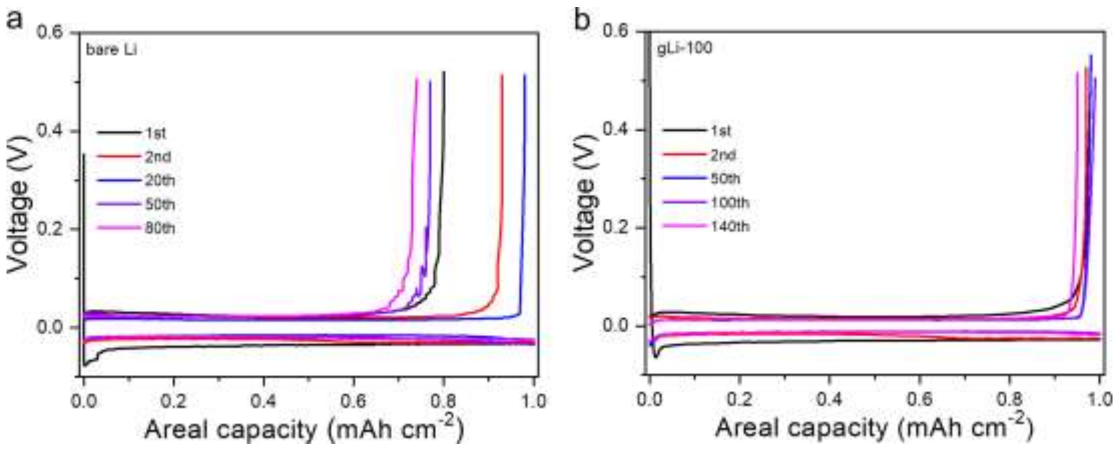

Figure S10. Selected Li stripping/plating curves from the a) bare-Li||Cu and b) gLi-100||Cu half-cells cycled at 1 mA cm^{-2} with a plating capacity of 1 mAh cm^{-2}, and a cut-off stripping voltage of 0.5 V.

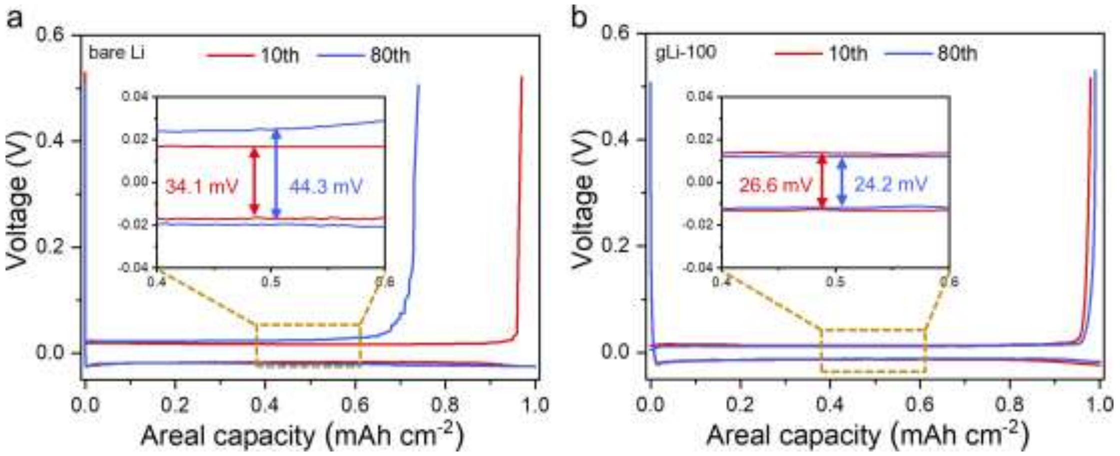

Figure S11. Stripping/plating curves from the a) bare-Li||Cu and b) gLi-100||Cu half-cells cycled at the 10th and 80th cycles. The inset graphs illustrate the magnified charge/discharge voltage hysteresis.

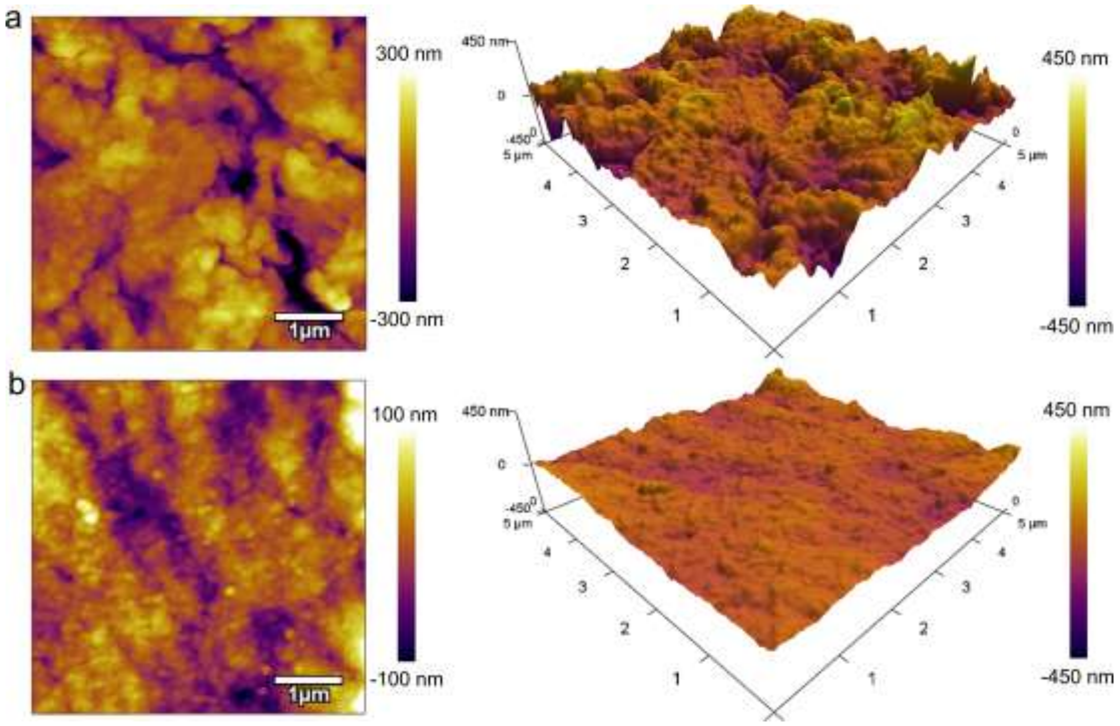

Figure S12. AFM top-view and 3D topograph of the a) bare-Li and b) gLi-100 electrodes disassembled from half-cells after 50 cycles.

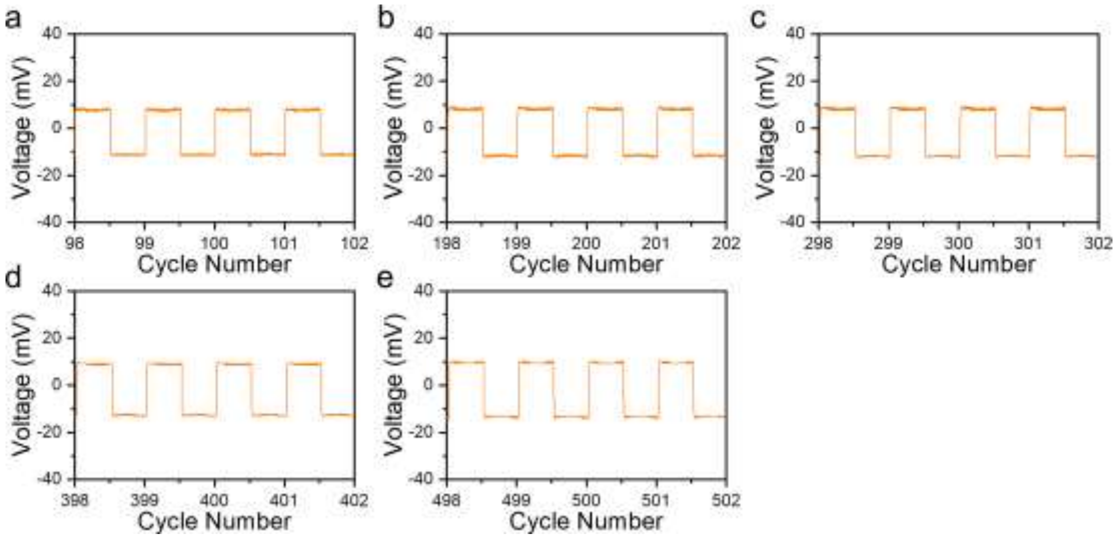

Figure S13. Magnified charge/discharge platforms at different states along the galvanostatic cycling of gLi-100 symmetrical cell with a fixed cycling capacity of 1 mAh cm^{-2} at 1 mA cm^{-2}.

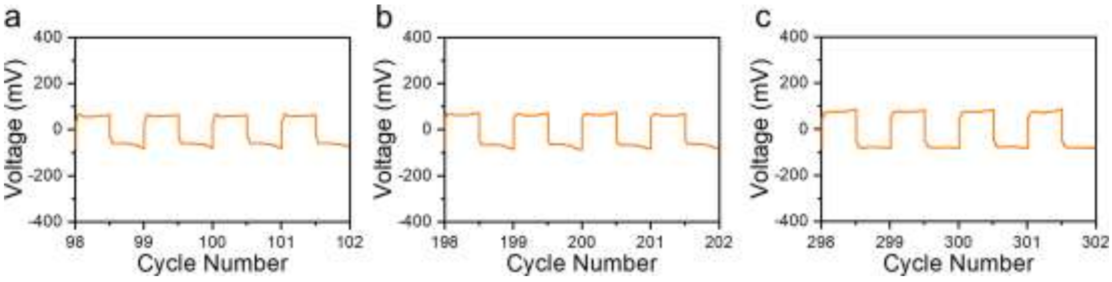

Figure S14. Magnified charge/discharge platforms at different states along the galvanostatic cycling of gLi-100 symmetrical cell with a fixed cycling capacity of 1 mAh cm^{-2} at 5 mA cm^{-2}.

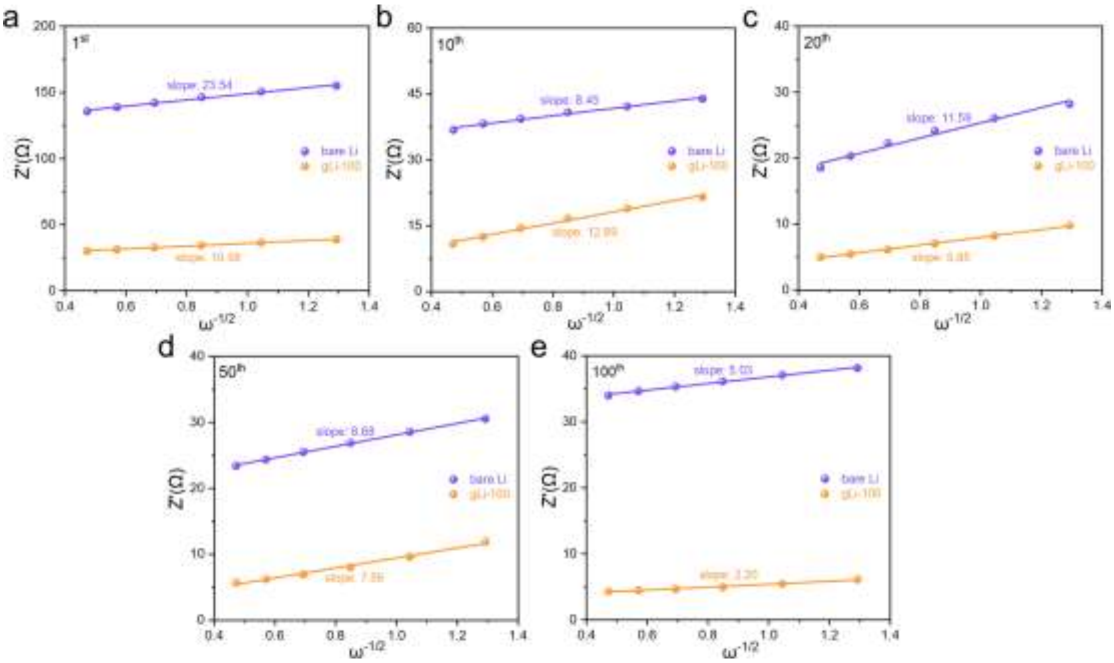

Fiure S15. The plots of Z′ to ω$^{-1/2}$ at different cycle status: a) 1st, b) 10th, c) 20th, d) 50th, e) 100th.

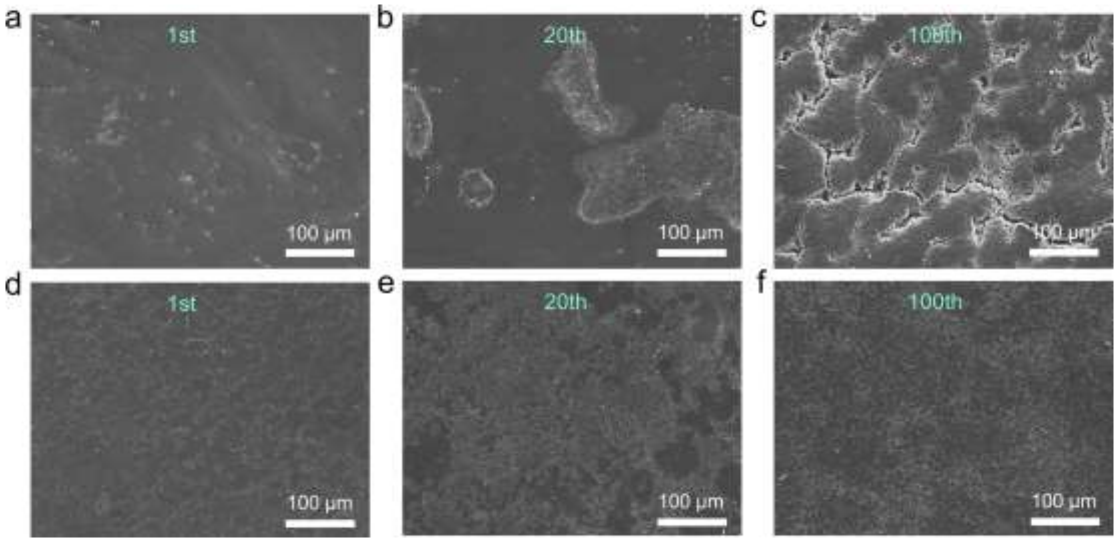

Figure S16. *Ex-situ* SEM images of the a-c) bare-Li and d-f) gLi-100 electrodes at different cycle status in symmetrical cells: after 1st cycle, after 20th cycle and after 100th cycle, respectively.

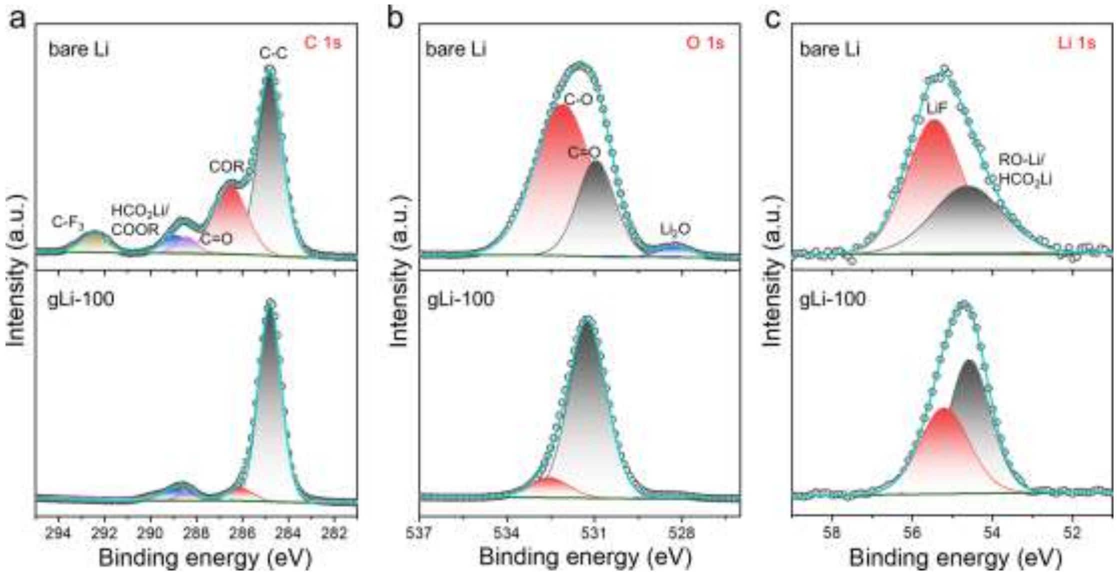

Figure S17. a) C 1s, b) O 1s and c) Li 1s XPS spectra of the bare Li (top) and gLi-100 (bottom) anodes after cycling.

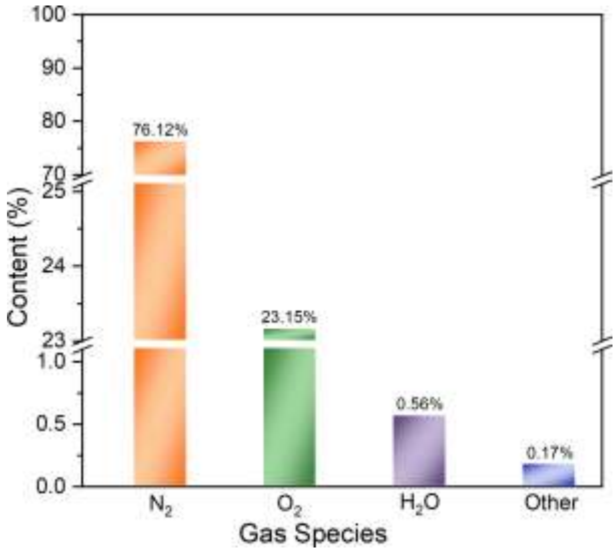

Figure S18. The gaseous content of the cylinder compressed air measured by mass spectroscopy.

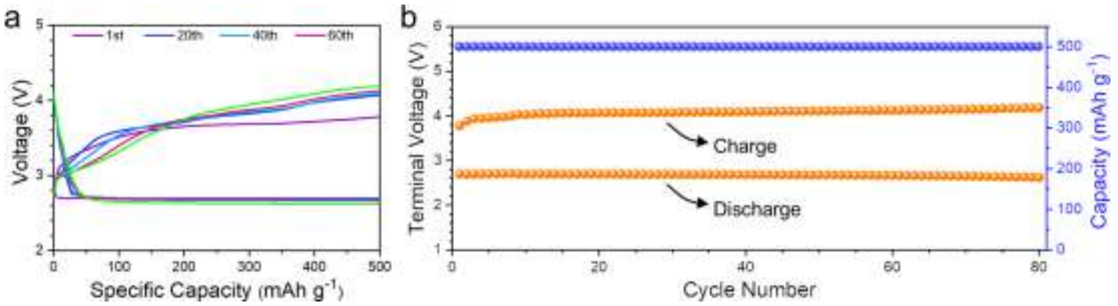

Figure S19. a) Serial discharge/charge profiles and b) long-term cycling performance of a gLi-100||Ru@CNT cell at the current density of 200 mA g^{-1} with the gLi-100 anode recycled from a former test lasting for 230 cycles.

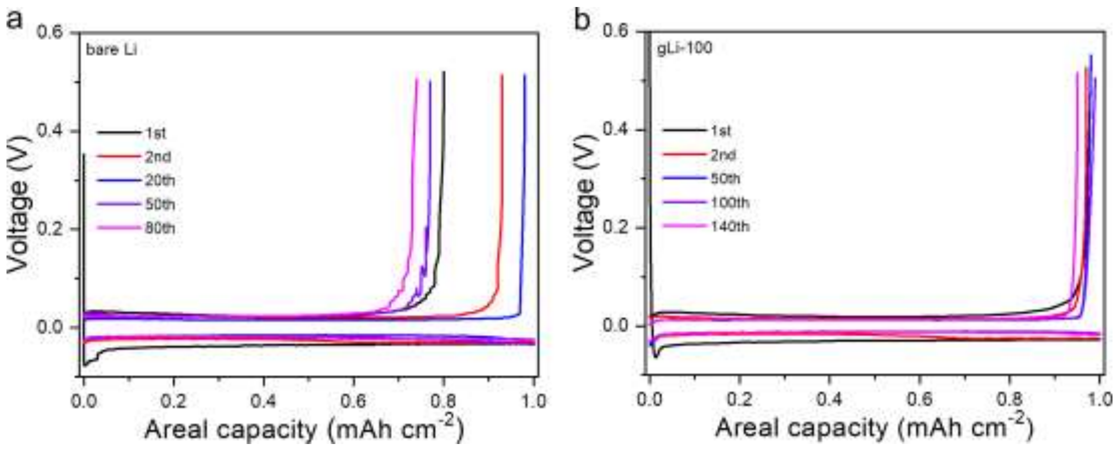

Figure S10. Selected Li stripping/plating curves from the a) bare-Li||Cu and b) gLi-100||Cu half-cells cycled at 1 mA cm^{-2} with a plating capacity of 1 mAh cm^{-2}, and a cut-off stripping voltage of 0.5 V.

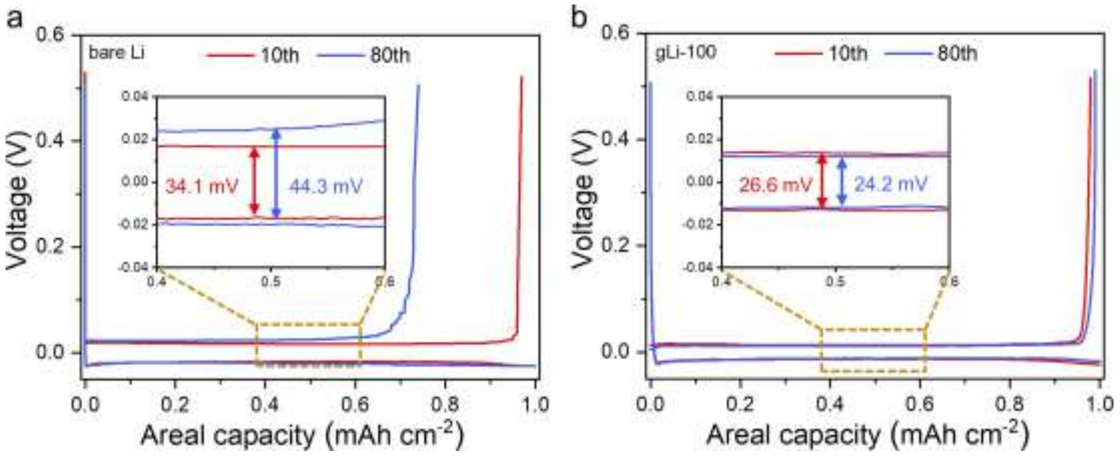

Figure S11. Stripping/plating curves from the a) bare-Li||Cu and b) gLi-100||Cu half-cells cycled at the 10th and 80th cycles. The inset graphs illustrate the magnified charge/discharge voltage hysteresis.

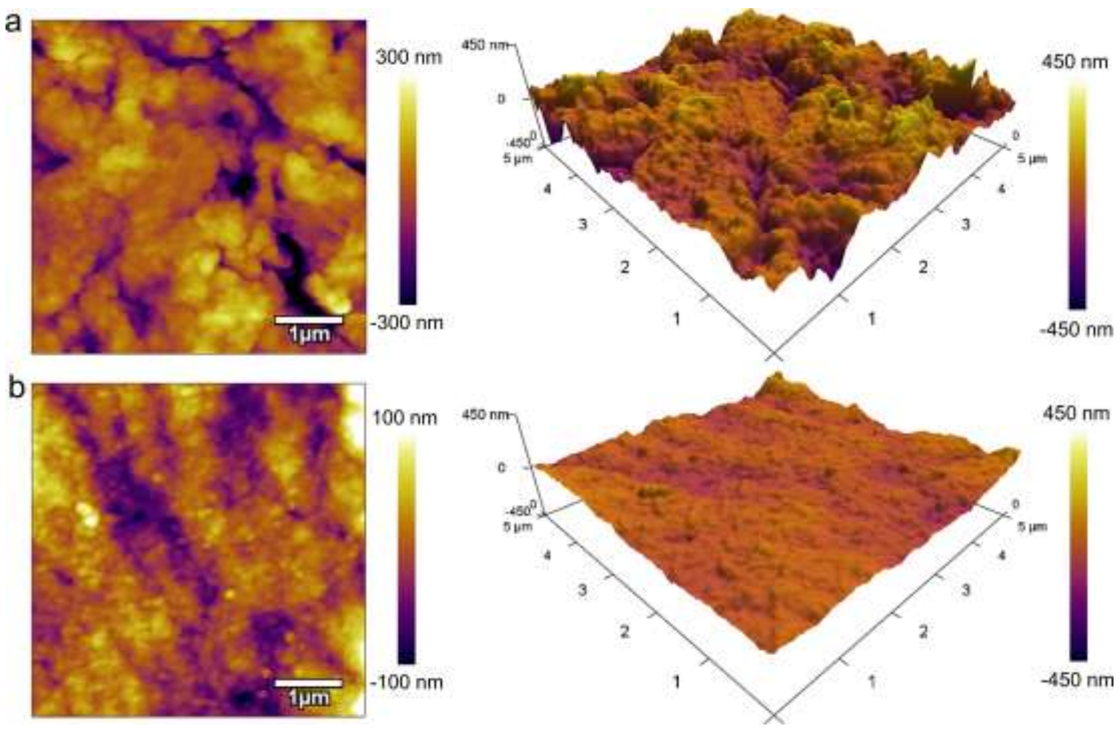

Figure S12. AFM top-view and 3D topograph of the a) bare-Li and b) gLi-100 electrodes disassembled from half-cells after 50 cycles.

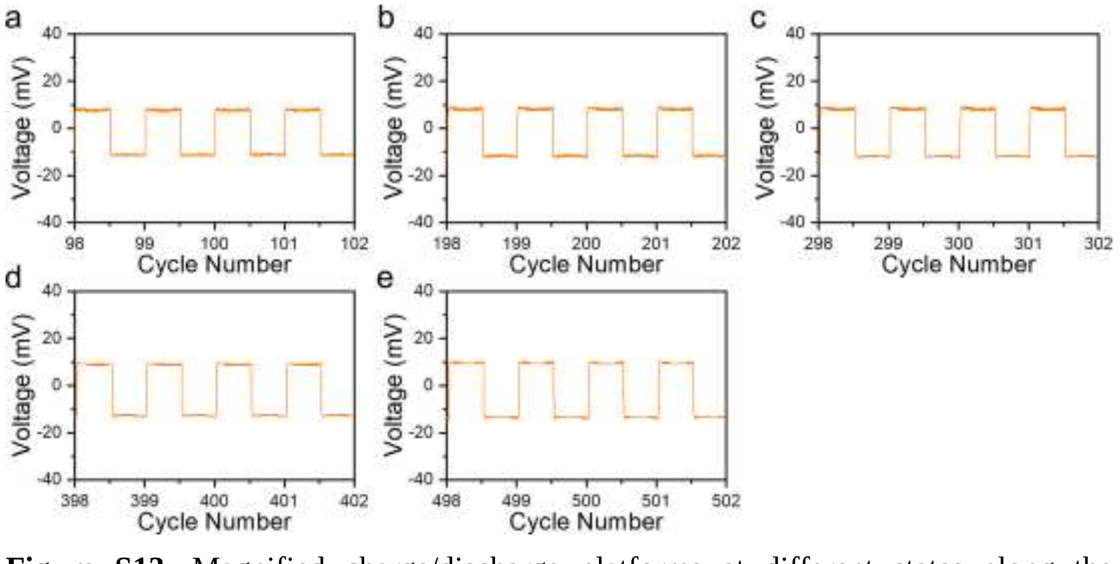

Figure S13. Magnified charge/discharge platforms at different states along the galvanostatic cycling of gLi-100 symmetrical cell with a fixed cycling capacity of 1 mAh cm^{-2} at 1 mA cm^{-2}.

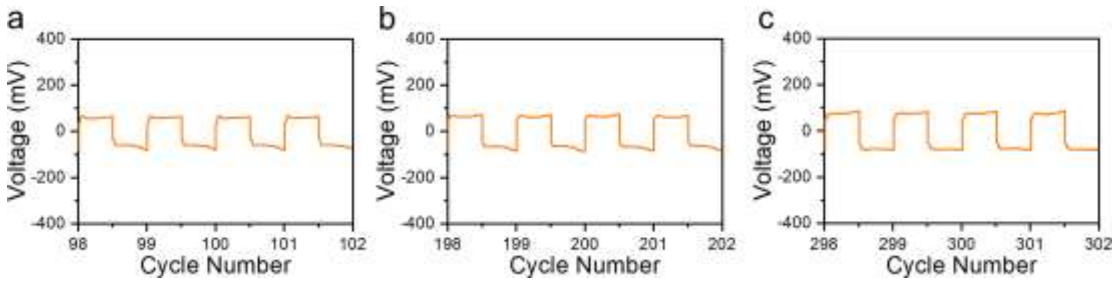

Figure S14. Magnified charge/discharge platforms at different states along the galvanostatic cycling of gLi-100 symmetrical cell with a fixed cycling capacity of 1 mAh cm^{-2} at 5 mA cm^{-2}.

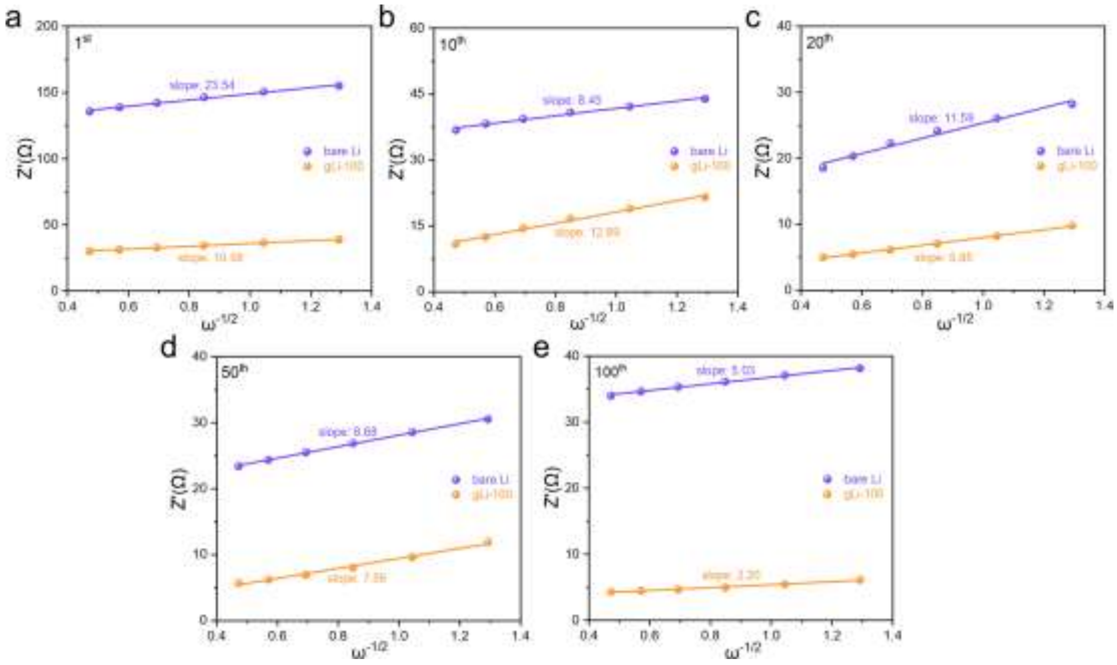

Fiure S15. The plots of Z′ to $\omega^{-1/2}$ at different cycle status: a) 1st, b) 10th, c) 20th, d) 50th, e) 100th.

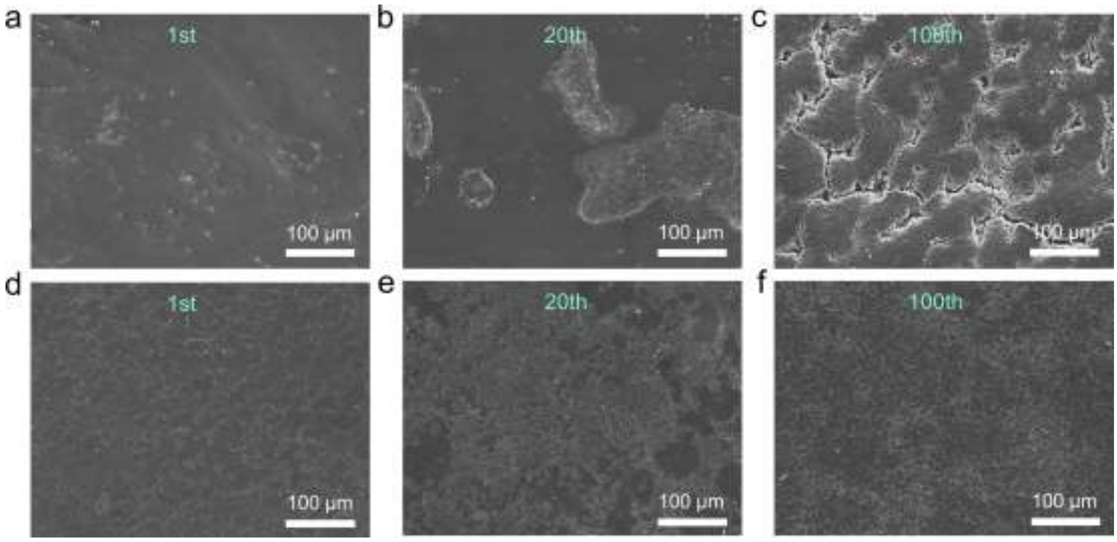

Figure S16. *Ex-situ* SEM images of the a-c) bare-Li and d-f) gLi-100 electrodes at different cycle status in symmetrical cells: after 1st cycle, after 20th cycle and after 100th cycle, respectively.

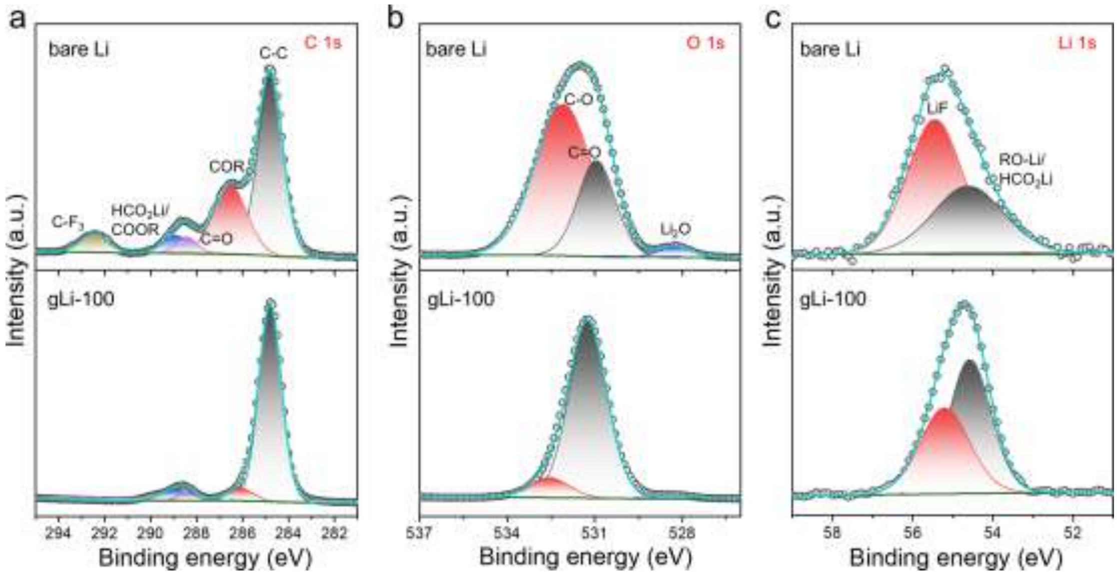

Figure S17. a) C 1s, b) O 1s and c) Li 1s XPS spectra of the bare Li (top) and gLi-100 (bottom) anodes after cycling.

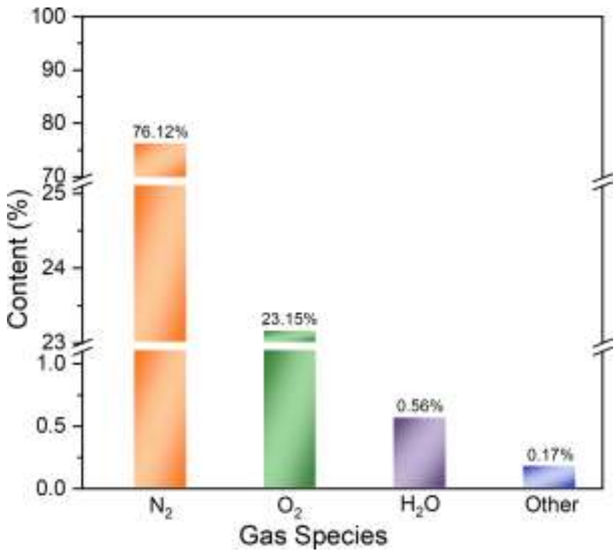

Figure S18. The gaseous content of the cylinder compressed air measured by mass spectroscopy.

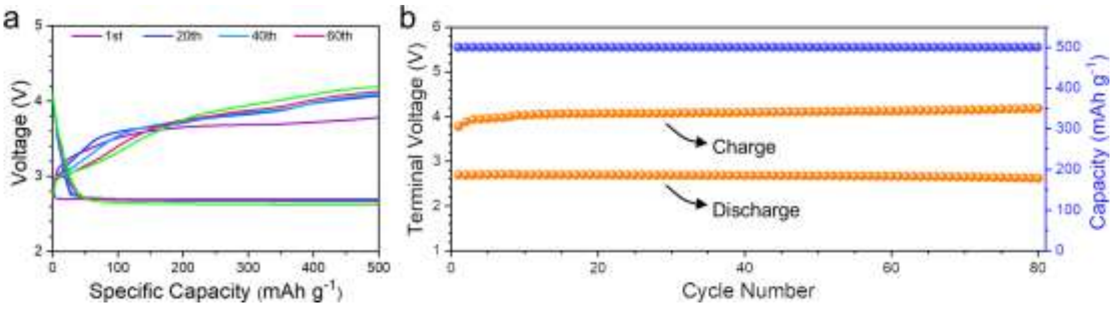

Figure S19. a) Serial discharge/charge profiles and b) long-term cycling performance of a gLi-100||Ru@CNT cell at the current density of 200 mA g^{-1} with the gLi-100 anode recycled from a former test lasting for 230 cycles.

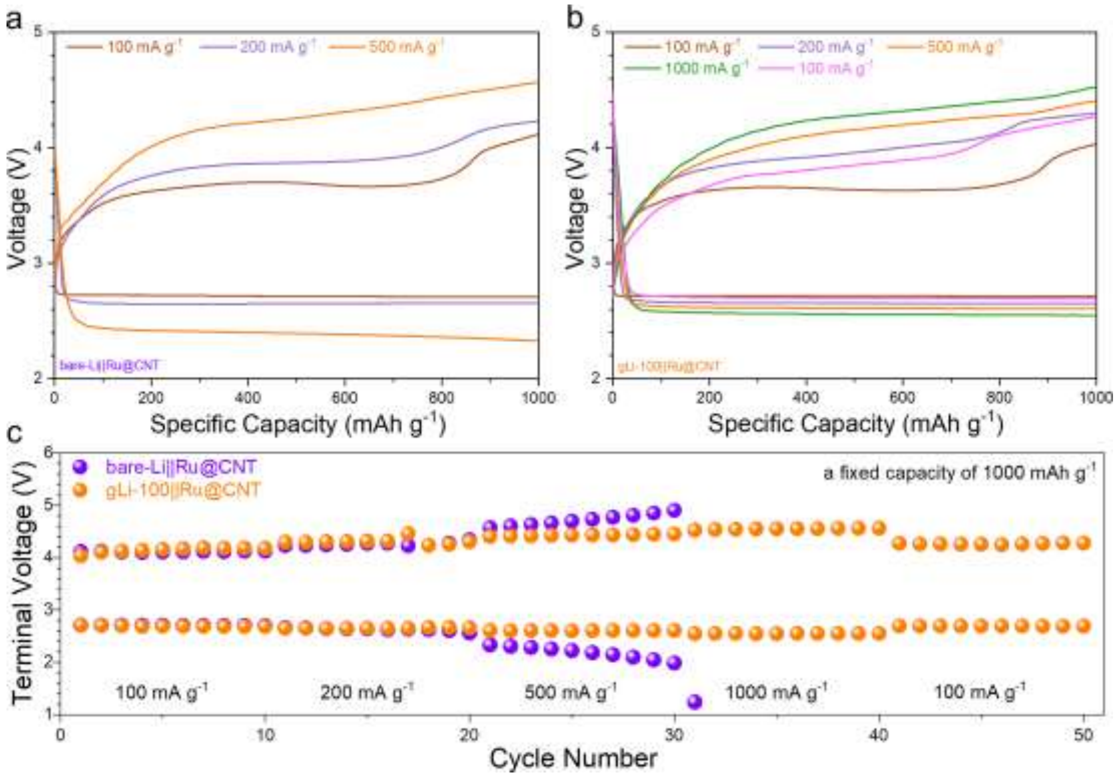

Figure S20. Serial discharge/charge profiles of the a) bare-Li||Ru@CNT and b) gLi-100||Ru@CNT cells cycled at various current density with a fixed cut-off capacity of 1000 mAh g^{-1}. Rate capabilities of the bare-Li||Ru@CNT and gLi-100||Ru@CNT cells cycled at varying current densities from 100 to 1000 mA g^{-1} with a fixed cut-off capacity of 1000 mAh g^{-1}.

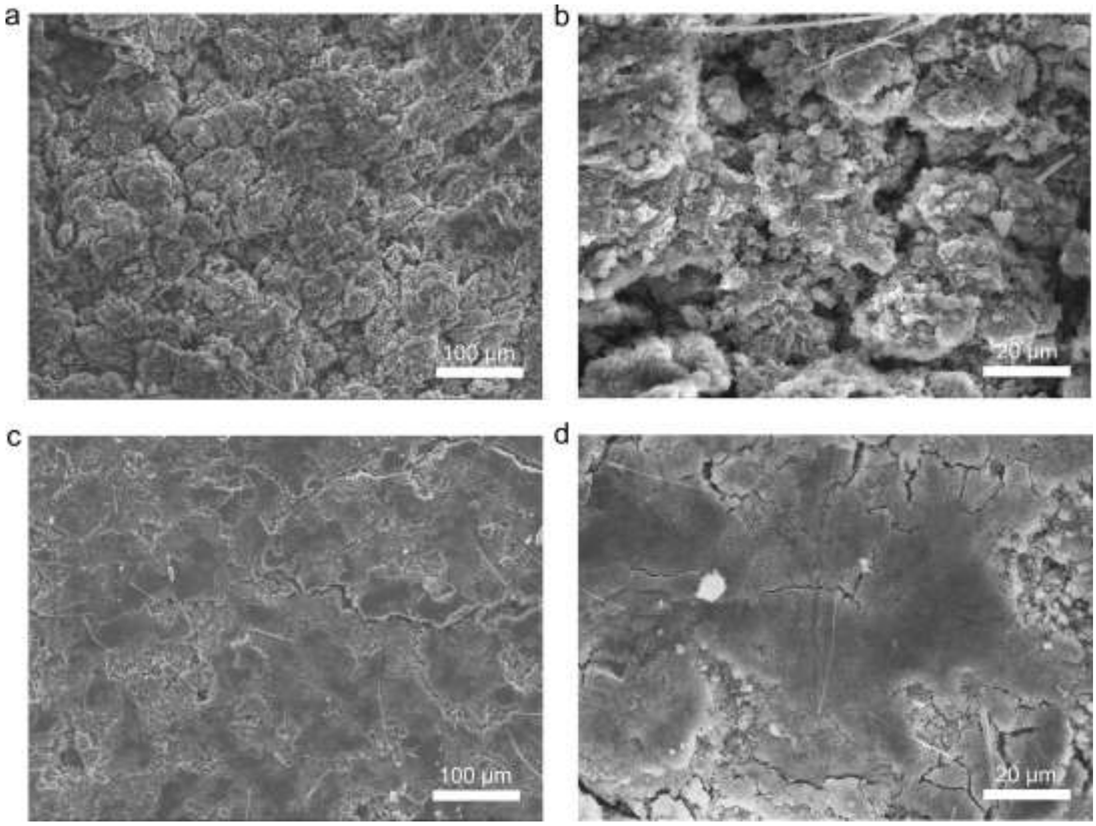

Figure S21. a, b) SEM images of the bare-Li anode disassembled from a Li-air battery after 50 cycles. c, d) SEM images of the gLi-100 anode disassembled from a Li-air battery after 120 cycles.

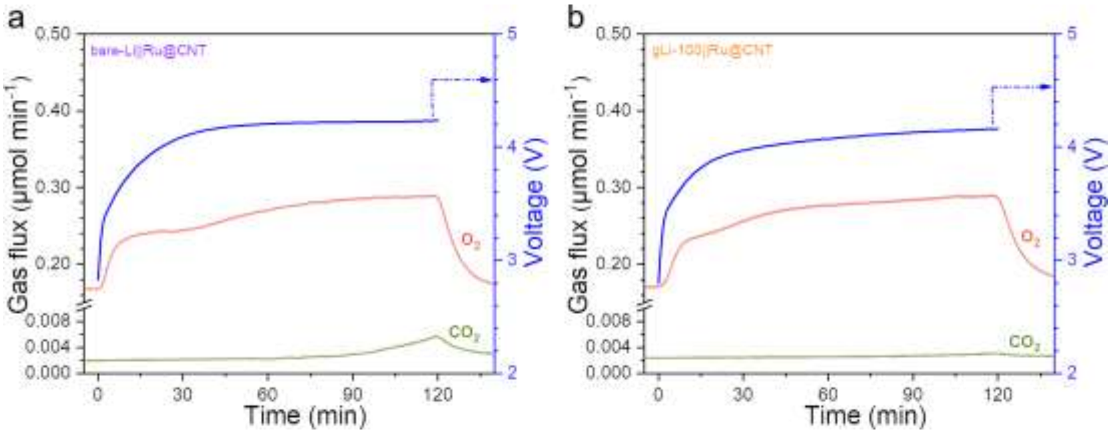

Figure S22. *In-situ* DEMS to quantify the gas evolution during the charging process of a) bare-Li||Ru@CNT and b) gLi-100||Ru@CNT cells.

Table S1. Values of Li^+ ionic conductance and diffusion constant used for modelling.[2-5]

	Ionic conductance (S cm^{-1})	Diffusion constant
electrolyte	1.2×10^{-2}	1×10^{-5}
SEI	1×10^{-6}	3×10^{-10}
graphene	1×10^{-2}	1×10^{-8}

Table S2. Statistics on the Young's modulus values acquired by AFM under the peak-force mode.

Total	Mean Value	Standard Deviation	Minimum	Median	Maximum
16	31.2375	4.34387	23.4	31.85	38.5

References

[1] P. Qi, Y. Huang, Y. Yao, Q. Li, Y. Lian, L. Lin, X. Wang, Y. Gu, L. Li and Z. Deng, *Appl. Surf. Sci.*, **2019**, 493, 81-86.

[2] G. Zheng, C. Wang, A. Pei, J. Lopez, F. Shi, Z. Chen, A. D. Sendek, H.-W. Lee, Z. Lu and H. Schneider, *ACS Energy Lett.*, **2016**, 1, 1247-1255.

[3] H. Chen, A. Pei, D. Lin, J. Xie, A. Yang, J. Xu, K. Lin, J. Wang, H. Wang and F. Shi, *Adv. Energy Mater.*, **2019**, 9, 1900858.

[4] F. J. Yang, Y. F. Huang, M. Q. Zhang and W. H. Ruan, *Polym.*, **2018,** 153, 438-444.

[5] C. Uthaisar and V. Barone, *Nano Lett.*, **2010**, 10, 2838-2842.
